# Early overnutrition in male mice negates metabolic benefits of a diet high in monounsaturated and omega-3 fats

**DOI:** 10.1038/s41598-021-93409-z

**Published:** 2021-07-07

**Authors:** Maria M. Glavas, Queenie Hui, Ian Miao, Fan Yang, Suheda Erener, Kacey J. Prentice, Michael B. Wheeler, Timothy J. Kieffer

**Affiliations:** 1 Department of Cellular and Physiological Sciences, University of British Columbia, Vancouver, BC Canada; 2 Department of Physiology, University of Toronto, Toronto, Canada; 3 Department of Advanced Diagnostics, Toronto General Hospital Research Institute, University Health Network, Toronto, Canada; 4 Department of Surgery, University of British Columbia, Vancouver, BC Canada; 5 School of Biomedical Engineering, University of British Columbia, Vancouver, BC Canada

**Keywords:** Physiology, Biomarkers, Endocrinology

## Abstract

Overconsumption of saturated fats promotes obesity and type 2 diabetes. Excess weight gain in early life may be particularly detrimental by promoting earlier diabetes onset and potentially by adversely affecting normal development. In the present study we investigated the effects of dietary fat composition on early overnutrition-induced body weight and glucose regulation in Swiss Webster mice, which show susceptibility to high-fat diet-induced diabetes. We compared glucose homeostasis between a high-fat lard-based (HFL) diet, high in saturated fats, and a high-fat olive oil/fish oil-based (HFO) diet, high in monounsaturated and omega-3 fats. We hypothesized that the healthier fat profile of the latter diet would improve early overnutrition-induced glucose dysregulation. However, early overnutrition HFO pups gained more weight and adiposity and had higher diabetes incidence compared to HFL. In contrast, control pups had less weight gain, adiposity, and lower diabetes incidence. Plasma metabolomics revealed reductions in various phosphatidylcholine species in early overnutrition HFO mice as well as with diabetes. These findings suggest that early overnutrition may negate any beneficial effects of a high-fat diet that favours monounsaturated and omega-3 fats over saturated fats. Thus, quantity, quality, and timing of fat intake throughout life should be considered with respect to metabolic health outcomes.

## Introduction

Obesity, resulting from overnutrition, is a main risk factor for type 2 diabetes. Overnutrition beginning in early life may be even more detrimental since exposure to an adverse nutritional environment may cause developmental malprogramming^1,2^. Childhood obesity can lead to earlier onset of type 2 diabetes and cardiovascular disease, resulting in reduced quality of life and shortened lifespan^3,4^. Although increased adiposity in early life and subsequent increased diabetes risk may be related to prenatal exposure to maternal diabetes or obesity^5^, overnutrition in the early postnatal period alone can also have long-term detrimental consequences for metabolic health. In rodent studies, early overnutrition can be induced by reducing litter size which, by reducing competition for maternal milk, results in increased body weight gain during the suckling period compared to control offspring, particularly the first two weeks of life when nutrient intake is solely from milk. We and others have shown that such early overnutrition results in long-term metabolic dysfunction^6–8^. In Swiss Webster mice, we previously observed that early overnutrition results in beta-cell dysfunction and increased diabetes incidence^6^. However, this could be modified by dietary fat content such that a low fat diet was protective but moderate to high levels of saturated fat increased diabetes incidence, even when limited to early life. Notably, the beta-cell dysfunction occurred in the absence of insulin resistance suggesting that elevated dietary fat intake and overnutrition in early life may directly impair beta-cell function and increase the risk of diabetes.

A Mediterranean diet has long been thought to promote metabolic and cardiovascular health^9,10^ and the fatty acid composition of this diet may play a key role in its beneficial effects. In place of high saturated fats that are typical of a western diet, the Mediterranean diet favors olive oil, which is high in the monounsaturated fat oleic acid, and fish with abundant omega-3 polyunsaturated fats. With respect to diabetes development, numerous studies suggest that the saturated fat palmitic acid is particularly detrimental to beta-cell function and promotes insulin resistance^11^. An increase in oleic acid intake in place of saturated fats has been shown to improve insulin sensitivity but effects of oleic acid on beta-cells have been less clear, with some studies suggesting protective effects and others suggesting lipotoxic effects^12–14^. Studies have also shown that DHA and EPA, omega-3 polyunsaturated fats abundant in fish oil, can attenuate lipotoxic effects of palmitic acid in beta-cell lines^15,16^ and improve metabolic health^17^. Of particular relevance, previous studies have found that a diet high in fish oil, administered after weaning, can improve glucose tolerance in rats exposed to early overnutrition^18,19^. Our goal in the present study was to examine whether early overnutrition-induced glucose dysregulation on a high fat diet can be improved by a healthier dietary fat composition favouring monounsaturated and omega-3 fats over saturated fats. A targeted plasma metabolomics approach was used to further explore effects of early overnutrition, diet, and hyperglycemia on lipid regulation.

## Results

### Diet differentially affects pup growth and tissue weights based on early overnutrition

**Figure 1:**
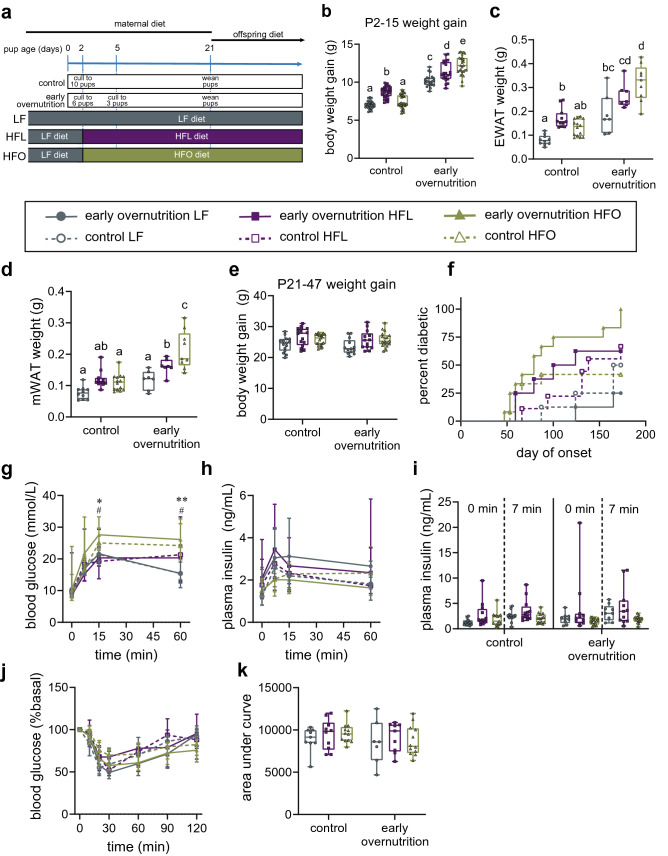
**(a)** All dams were maintained on low fat (LF) diet through pregnancy and until 2 days post delivery. At postnatal day (P) 2, control litters were culled to 10 pups and early overnutrition litters were initially culled to 6 pups and then to 3 pups at P5. Dams were either maintained on LF diet throughout lactation or switched to lard-based high-fat (HFL) diet or an olive oil and fish oil-based high-fat (HFO) diet at P2, with offspring weaned onto the same diet at postnatal day P21 to generate LF, HFL, and HFO offspring, respectively. Body weight gain from **(b)** P2 to 15 with n = 21–27 pups per group from 5 to 7 litters, **(c)** epididymal white adipose tissue (EWAT) weight at P21, and **(d)** mesenteric white adipose tissue weight (mWAT) at P21 (n and litter size as per Table 3). Differing letters denote statistically significant differences between groups, p < 0.05. **(e)** P21 to 47 body weight gain, n = 8–21 per group from 5–9 litters. **(f)** Diabetes incidence to P173, with early overnutrition HFO > early overnutrition LF (p = 0.0003), control HFO (p = 0.0099), control HFL (p = 0.0223), and control LF (p = 0.0039) by log-rank test; n = 8–12 from 3 to 9 litters. Blood glucose **(g)** and plasma insulin **(h)** responses to oral glucose gavage at P59; n = 9–15 from 5–7 litters. **(i)** Individual plasma levels at 0 and 7 min are shown for easier comparison among groups. Blood glucose **(j)** and area under the curve **(k)** in response to intraperitoneal insulin injection at P64; n = 9–12 from 3 to 12 litters. *Early overnutrition HFO > early overnutrition LF, control HFL, and control LF, p’s < 0.05. ^#^Control HFO > early overnutrition LF, p < 0.05. **Early overnutrition HFO > early overnutrition LF, p < 0.005. *LF* low-fat diet; *HFL* high-fat lard-based diet; *HFO* high-fat olive oil/fish oil-based diet.

All dams consumed low-fat (LF) diet during pregnancy until two days post-delivery, designated as postnatal day (P) 2 (study design in Fig. 1a). Litters were culled to either 3 pups to generate early overnutrition offspring or 10 pups for control offspring. At P2, dams either remained on LF diet or were switched to either a high-fat (45 kcal% fat) lard-based (HFL) or high-fat (45 kcal% fat) olive oil/fish oil-based (HFO) diet. Diet composition and fatty acid profiles of the three diets are in Tables 1 and 2. Caloric content was matched between HFL and HFO diets. Offspring were weaned onto the same diet as the dams. As expected, early overnutrition resulted in greater body weight gain on all diets compared to control pups (Suppl Fig. S1a). Since these mouse pups begin to consume some solid food at P16, body weight gain from P2 to 15 (Fig. 1b) reflects nutritional intake only from maternal milk. Post-hoc analysis (significant group × diet interaction, p < 0.0001, all two-way ANOVA details in Supplementary Table S1) revealed that early overnutrition pups of HFL-consuming dams had increased body weight gain compared to LF pups (p < 0.0001). Unexpectedly, however, early overnutrition HFO pups gained even more weight than HFL pups (p = 0.025). In contrast, control pups showed the expected attenuation of body weight gain in the HFO group relative to HFL (p < 0.0001). Indeed, higher adiposity (Fig. 1c,d, Table 3), particularly with mesenteric fat pad weight, was observed in early overnutrition HFO pups relative to HFL suggesting a greater adipogenic effect, although this difference was diminished when normalized to body weight (Table 3). Blood glucose at P21 was higher (main effect of diet, p < 0.0001) in HFL and HFO relative to LF pups (Table 3) whereas insulin was higher overall (main effect of group, p = 0.036) in early overnutrition compared to control pups, suggesting early development of glucose dysregulation. Liver weight (Table 3) was also greater in early overnutrition compared to control pups (main effect of diet, p < 0.0001) and elevated in HFL and HFO pups relative to LF (main effect of diet, p = 0.001); however, these differences were abolished when normalized to body weight.

**Table 1:** Diet composition.

	LF diet (10 kcal% fat)		HFL diet (45 kcal% fat)		HFO diet (45 kcal% fat)							
	g%	kcal%	g%	kcal%	g%	kcal%						
Protein	19.2	20	24	20	24	20						
Carbohydrate	67.3	70	41	35	41	35						
Fat	4.3	10	24	45	24	45						
Energy density	3.85 kcal/g		4.7 kcal/g		4.7 kcal/g							

Ingredient	g	g	kcal	kcal	g	g	kcal	kcal	g	g	kcal	kcal
		%		%		%		%		%		%
Casein, 80 mesh	200	19.0	800	19.7	200	23.3	800	19.7	200	23.3	800	19.7
L-cystine	3	0.3	12	0.3	3	0.3	12	0.3	3	0.3	12	0.3
Corn Starch	315	29.9	1260	31.1	72.8	8.5	291	7.2	72.8	8.5	291	7.2
Maltodextrin	35	3.3	140	3.5	100	11.7	400	9.9	100	11.7	400	9.9
Sucrose	350	33.2	1400	34.5	172.8	20.1	691	17.0	172.8	20.1	691	17.0
Cellulose, BW200	50	4.7	0	0.0	50	5.8	0	0	50	5.8	0	0
Soybean oil	25	2.4	225	5.5	25	2.9	225	5.5	25	2.9	225	5.5
Lard	20	1.9	180	4.4	177.5	20.7	1598	39.4	20	2.3	180	4.4
Olive Oil	0	0.0	0	0.0	0	0.0	0	0.0	130	15.2	1170	28.8
Menhaden (fish) oil	0	0	0	0	0	0	0	0	27.5	3.2	248	6.1
Mineral mix	10	0.9	0	0	10	1.2	0	0	10	1.2	0	0
Calcium Phosphate, dibasic	13	1.2	0	0	13	1.5	0	0	13	1.5	0	0
Calcium carbonate	5.5	0.5	0	0	5.5	0.6	0	0	5.5	0.6	0	0
Potassium citrate	16.5	1.6	0	0	16.5	1.9	0	0	16.5	1.9	0	0
vitamin mix	10	0.9	40	1.0	10	1.2	40	1.0	10	1.2	40	1.0
Choline tartrate	2	0.2	0	0	2	0.2	0	0	2	0.2	0	0
Total	1055	100	4057	100	858.2	100	4057	100	858	100	4057	100

*LF* low-fat diet (#D12450B). *HFL* high-fat lard-based diet (#D12451). *HFO* custom olive oil and fish (menhaden) oil-based high-fat diet (#D10072204). All diets and composition values provided by Research Diets, Inc. (New Brunswick, NJ).

**Table 2:** Fatty acid profile of diets.

Fatty acid	LF diet		HFL diet		HFO diet	
	g	g%	g	g%	g	g%
C14, Myristic	0.2	0.02	1.6	0.19	2.1	0.24
C16, Palmitic	7.2	0.68	43.2	5.03	26.7	3.11
C16:1, Palmitoleic	0.8	0.08	6.7	0.78	4.9	0.57
C18, Stearic	3.6	0.34	24.6	2.87	7.5	0.87
C18:1, Oleic	14.3	1.36	79	9.21	109.4	12.75
C18:2, Linoleic*	15.1	1.43	28.6	3.33	32.8	3.82
C18:3,Linolenic^†^	2.2	0.21	3.7	0.43	3.3	0.38
C20:4, Arachidonic*	0.3	0.03	3	0.35	0.8	0.09
C20:5, EPA^†^	0	0	0	0	4.2	0.49
C22:6, DHA^†^	0	0	0	0	3	0.35
Total	43.7	4.3	191.4	24	199.2	24
Saturated fats (g)	11		69.4		37.1	
Monounsaturated fats (g)	15.2		86.7		115.3	
Polyunsaturated fats (g)	17.6		35.4		46.2	
Saturated fats (%)	25.1		36.3		18.6	
Monounsaturated fats (%)	34.7		45.3		57.9	
Polyunsaturated fats (%)	40.2		18.5		23.2	
omega-6: omega-3 ratio	7		8.5		3	

*LF* low-fat diet (#D12450B). *HFL* high-fat lard-based diet (#D12451). *HFO* olive oil and fish (menhaden) oil-based high-fat diet (#D10072204). All diets and values provided by Research Diets, Inc. (New Brunswick, NJ).

*Omega-6.

^†^Omega-3.

**Table 3:** Tissue weights and measures in non-fasted postnatal day 21 mice.

Tissue/measure	Control LF	Control HFL	Control HFO	Early over-nutrition LF	Early over-nutrition HFL	Early over-nutrition HFO	Group effect	Diet effect	Group × diet
n	10	11	14	7	7	9			
# litters	5	5	7	7	7	9			
Body weight (g)	16.1^a^	19.4^bc^	17.7^ab^	18.7^bc^	21.1cd	22.3^d^	F = 53.54	F = 17.41	F = 4.85
	(15.8–16.4)	(18.1–20.6)	(16.2–19.2)	(18.4–19.8)	(20.8–21.8)	(20.9–24.1)	p < 0.001	p < 0.001	*p = 0.012
Glucose (mmol/L)	8.2	9.7	9.7	8.7	9.3	10.3	F = 0.392	F = 11.26	F = 2.34
	(8.1–8.8)	(9.5–10.5)	(9.1–10.1)	(8.5–9.0)	(8.5–10.4)	(9.6–10.9)	p = 0.534	^†^p < 0.001	p = 0.107
Insulin (ng/mL)	1.26	1.66	0.97	1.60	1.66	2.06	F = 4.65	F = 1.27	F = 2.40
	(0.89–1.58)	(1.13–2.85)	(0.76–1.44)	(1.09–1.98)	(1.38–3.82)	(1.25–4.70)	^§^p = 0.036	p = 0.289	p = 0.101
EWAT (mg/g bw)	4.809	8.430	7.089	8.920	11.950	14.140	F = 82.68	F = 14.50	F = 2.50
	(4.137–5.468)	(7.747–9.332)	(6.217–8.881)	(7.642–11.06)	(11.11–13.13)	(12.82–15.74)	^§^p < 0.001	^†^p < 0.001	P = 0.092
mWAT (mg/g bw)	4.727	6.281	5.734	6.706	7.799	9.228	F = 30.96	F = 13.88	F = 2.84
	(3.703–5.382)	(5.403–6.320)	(5.542–6.703)	(5.374–7.140)	(7.239–8.063)	(7.848–9.892)	^§^p < 0.001	^†^p < 0.001	p = 0.068
rpWAT (g)	0.014^a^	0.040^b^	0.027^b^	0.034^ab^	0.063^c^	0.075^c^	F = 59.65	F = 21.41	F = 6.58
	(0.013–0.020)	(0.035–0.044)	(0.021–0.041)	(0.029–0.042)	(0.052–0.073)	(0.063–0.088)	p < 0.001	p < 0.001	*p = 0.003
rpWAT (mg/g bw)	0.895 ^a^	1.851 ^bc^	1.544 ^ab^	1.971 ^ab^	2.953 cd	3.455 ^d^	F = 35.46	F = 14.14	F = 3.32
	(0.798–1.210)	(1.743–2.184)	(1.197–2.072)	(1.520–2.145)	(2.535–3.400)	(3.048–3.609)	p < 0.001	p < 0.001	*p = 0.044
Liver (g)	0.847	1.041	0.945	1.024	1.075	1.126	F = 31.46	F = 8.75	F = 2.64
	(0.807–0.897)	(0.944–1.064)	(0.863–1.019)	(0.976–1.050)	(1.026–1.148)	(1.120–1.278)	^§^p < 0.001	^†^p = 0.001	p = 0.081
Liver (mg/g bw)	52.46	51.67	53.70	54.74	51.00	52.11	F = 1.02	F = 2.22	F = 0.35
	(51.62–56.47)	(50.41–53.47)	(52.19–54.63)	(51.69–55.83)	(48.11–52.29)	(50.07–53.20)	p = 0.317	p = 0.119	p = 0.704

Values represent median (interquartile range). Note, two glucose measures missing from control LF pups and one missing insulin sample from early overnutrition LF group. LF: 10 kcal% fat diet; HFL: 45 kcal% fat lard-based diet; *HFO* 45 kcal% fat olive oil/fish oil-based diet; *eWAT* epididymal white adipose tissue; *mWAT* mesenteric white adipose tissue; *rpWAT* retroperitoneal white adipose tissue; *bw* body weight. Analysis: two-way ANOVA, Tukey’s correction for multiple comparisons.

*Significant group × diet interaction effect, with differences (p < 0.05) denoted by differing letter superscripts per condition. Significant main effects indicated only in absence of interaction effect.

^§^Main effect of group, early overnutrition > control.

^†^Main effect of diet, HFL = HFO > LF.

### Diet and early overnutrition differentially affect tissue weights and circulating hormones

Following weaning, body weight gain no longer differed between control and early overnutrition offspring (Fig. 1e, Suppl. Fig. S1b). Although we did not measure food intake in this study, this is consistent with our previous observations^6^ that increased food intake and accelerated weight gain is limited to the suckling period in early overnutrition pups. However, as expected, HFL- and HFO- diet fed mice gained more weight than LF diet-fed mice (main effect of diet, p = 0.001; HFO = HFL > LF) and early overnutrition mice overall maintained higher body weight and adiposity relative to controls (main diet effects), with higher mWAT, but not EWAT or rpWAT, persisting even when normalized to body weight (Table 4). In addition, insulin and leptin levels were elevated in control HFO mice compared to all other groups. Leptin is typically proportional to body fat, but this is not the case with control HFO mice which did not have higher adiposity (Table 4). Liver weight was overall higher in early overnutrition relative to control mice, even when normalized to body weight (Table 4), and higher in HFL and HFO compared to LF, although only the HFL elevation persisted when normalized to body weight.

**Table 4:** Tissue weights and measures in fasted postnatal day 70 mice.

Tissue/measure	Control LF	Control HFL	Control HFO	Early Over-nutrition LF	Early Over-nutrition HFL	Early Over-nutrition HFO	Group effect	Diet effect	Group × diet
n	7	7	9	6	5	6			
# litters	3	3	5	3	3	6			
Body weight (g)	46.9	52.3	50.9	50.3	55.1	54.1	7.10	5.58	0.05
	(45.3–49.2)	(50.1–53.5)	(47.9–53.2)	(46.6–53.7)	(50.6–58.5)	(49.7–57.8)	^§^p = 0.012	^†^p = 0.008	p = 0.954
Glucose (mmol/L)	8.8 ^a^	33.3 ^b^	8.7 ^a^	8.3 ^a^	13.4 ^ab^	26.4 ^b^	F = 0.03	F = 9.52	F = 8.11
	(8.3–10.1)	(8.6–33.3)	(7.8–13.5)	(7.1–9.0)	(10.2–22.7)	(12.2–29.2)	p = 0.8715	p < 0.001	*p = 0.001
Insulin (ng/mL)	1.29 ^a^	0.68 ^a^	4.94 ^b^	1.32 ^a^	1.60 ^a^	1.04 ^a^	F = 6.50	F = 6.39	F = 5.77
	(1.02–2.72)	(0.43–2.65)	(3.40–7.40)	(0.64–1.59)	(0.47–2.97)	(0.78–2.18)	p = 0.016	p = 0.005	*p = 0.008
Leptin (ng/mL)	12.51 ^a^	7.92 ^a^	47.94 ^b^	21.37 ^a^	26.97 ^a^	21.28 ^a^	F = 0.54	F = 6.14	F = 10.84
	(9.56–21.62)	(5.78–33.06)	(40.05–58.53)	(15.22–35.16)	(21.44–33.40)	(8.03–29.85)	P = 0.468	P = 0.006	*P < 0.001
EWAT (g)	0.926	1.016	0.986	1.033	1.347	1.066	F = 4.41	F = 4.25	F = 1.30
	(0.816–0.980)	(0.962–1.215)	(0.964–1.154)	(0.938–1.174)	(1.086–1.660)	(0.740–1.376)	^§^p = 0.043	^‡^p = 0.023	p = 0.287
EWAT (g/100 g bw)	1.862	1.919	2.011	2.117	2.428	2.038	F = 1.90	F = 3.28	F = 2.11
	(1.804–2.059)	(1.853–2.298)	(1.886–2.264)	(1.912–2.189)	(2.148–2.842)	(1.400–2.251)	p = 0.1770	p = 0.050	p = 0.136
mWAT (g)	0.667	0.715	1.008	0.982	1.558	1.160	F = 11.94	F = 1.70	F = 1.25
	(0.609–0.825)	(0.625–0.940)	(0.889–1.140)	(0.848–1.147)	(0.905–1.672)	(0.993–1.404)	^§^p = 0.002	p = 0.197	p = 0.298
mWAT	1.394	1.419	2.042	2.007	2.618	2.214	F = 10.16	F = 0.73	F = 1.78
(g/100 g bw)	(1.347–1.646)	(1.229–1.757)	(1.810–2.157)	(1.668–2.341)	(1.787–2.970)	(1.746–2.419)	^§^p = 0.001	p = 0.491	p = 0.184
rpWAT (g)	0.217	0.235	0.206	0.248	0.247	0.296	F = 5.83	F = 0.28	F = 0.75
	(0.167–0.279)	(0.182–0.252)	(0.155–0.229)	(0.228–0.291)	(0.214–0.322)	(0.234–0.351)	^§^p = 0.021	p = 0.759	p = 0.479
rpWAT (g/100 g bw)	0.451	0.459	0.384	0.502	0.448	0.559	F = 3.94	F = 0.20	F = 0.74
	(0.369–0.566)	(0.371–0.477)	(0.330–0.453)	(0.458–0.543)	(0.410–0.563)	(0.437–0.619)	p = 0.0554	p = 0.819	p = 0.483
Liver (g)	2.018	2.849	2.297	2.465	2.787	3.245	F = 8.46	F = 6.62	F = 0.30
	(1.934–2.142)	(2.550–2.890)	(2.210–3.036)	(2.173–2.762)	(2.541–3.623)	(2.540–3.582)	^§^p = 0.006	^†^p = 0.004	p = 0.744
Liver (g/100 g bw)	4.165	5.455	4.951	4.883	5.454	6.030	F = 4.55	F = 4.02	F = 0.32
	(4.062–4.740)	(5.007–5.840)	(4.441–5.763)	(4.555–5.440)	(4.680–6.429)	(5.030–6.395)	^§^p = 0.040	^‡^p = 0.027	p = 0.727

Values represent median (interquartile range). LF: 10 kcal% fat diet; HFL: 45 kcal% fat lard-based diet; HFO: 45 kcal% fat olive oil/fish oil-based diet; *eWAT* epididymal white adipose tissue; *mWAT* mesenteric white adipose tissue; *rpWAT* retroperitoneal white adipose tissue; *bw* body weight. Analysis: two-way ANOVA, Tukey’s correction for multiple comparisons.

*Significant group × diet interaction effect, with differences (p < 0.05) denoted by differing letter superscripts per condition. Significant main effects indicated only in absence of interaction effect.

^§^Main effect of group, early overnutrition > control.

^†^Main effect of diet, HFL = HFO > LF.

^‡^Main effect of diet, HFL > HFO = LF. For leptin and insulin measures, n = 6 per group.

### Diabetes incidence is affected by early overnutrition and diet

Diabetes (≥ 20 mmol/L after 4 h fast) incidence was assessed to 6 months of age (Fig. 1f). Surprisingly, in early overnutrition mice HFO diet did not reduce diabetes incidence compared to HFL diet, but in fact all (12/12) early overnutrition HFO mice developed diabetes, compared to 63% (5/8) on HFL diet. The lowest incidence was observed with early overnutrition LF mice, where 25% (2/8) developed diabetes. In contrast, and as we had expected, HFO diet did reduce diabetes incidence in control mice, with 42% (5/12) of control HFO mice developing diabetes compared to 67% (6/9) of HFL control mice. However, this difference did not reach statistical significance. The diabetes incidence in control HFO mice was even lower than control mice on LF diet, which had a 50% incidence (4/8) rate, perhaps related to the higher relative carbohydrate level in the LF diet.

### Glucose regulation and beta-cell mass in adults

**Figure 2:**
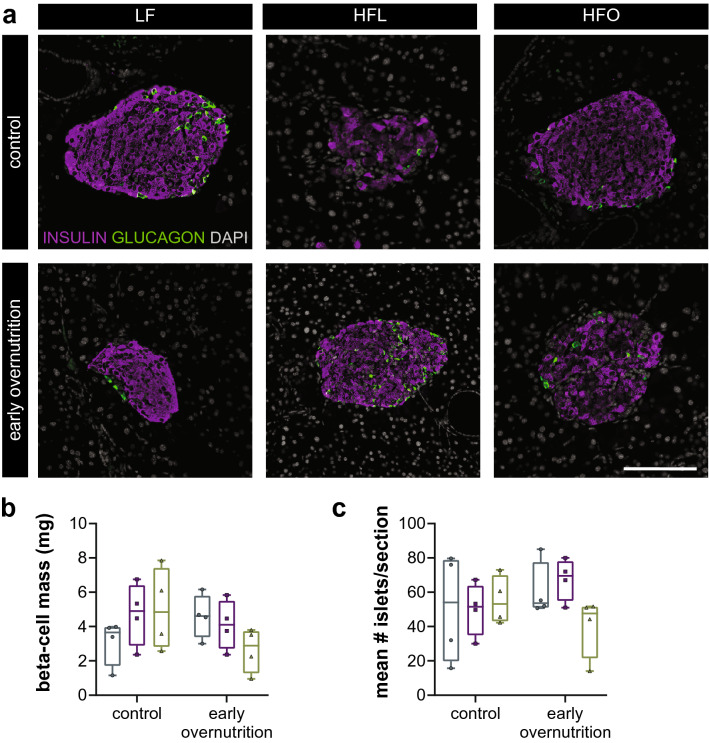
**(a)** Representative pancreas sections from postnatal day 70 Swiss Webster mice stained for insulin (magenta), glucagon (green), and DAPI (white). **(b)** Beta-cell mass and **(c)** mean # islets per section averaged over the sections per mouse. *LF* low-fat diet; *HF* high-fat, lard-based diet; *HFO* high-fat, olive oil-based diet. Scale bar = 100 μm.

During an oral glucose tolerance test at P59 (Fig. 1g–i), both early overnutrition and control HFO offspring had higher glucose excursions relative to their LF-fed counterparts, although insulin responses were not significantly different. No glucose differences were seen among groups during an insulin tolerance test (Fig. 1j,k), suggesting similar insulin sensitivity. Immunohistochemistry of pancreas with insulin and glucagon did not reveal any distinct morphological differences among treatment groups (Fig. 2a). However, as we have previously observed^6^, hyperglycemic mice tended to exhibit heterogeneous insulin staining such that insulin immunoreactivity appeared reduced in some cells, as seen in the control HFL and early overnutrition HFO islets in Fig. 2a. However, overall beta-cell mass and number of islets, measured at P70, did not differ among groups (Fig. 2b,c).

### Plasma phosphatidylcholines are altered by early overnutrition and diet

**Figure 3:**
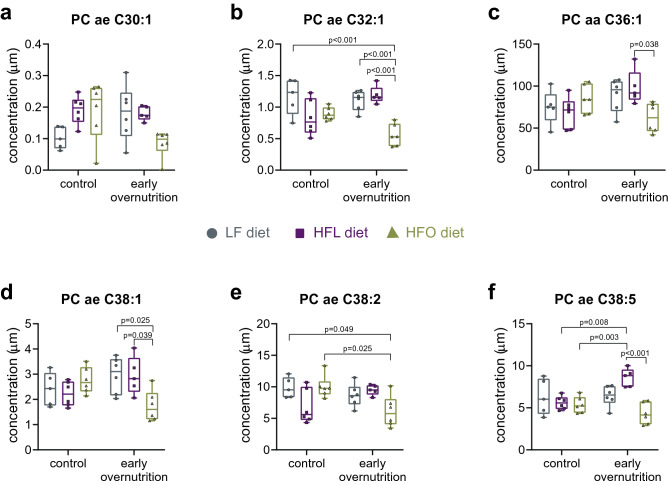
**(a–f)** Phosphatidylcholine metabolites exhibiting significant group by diet interactions determined by ANOVA Simultaneous Component Analysis and Tukey’s post hoc correction for multiple comparisons. *LF* low-fat diet; *HFL* high-fat lard-based diet; *HFO* high-fat olive oil/fish oil-based diet. Early overnutrition LF: n = 6, 3 litters; early overnutrition HFL: n = 5, 3 litters; early overnutrition HFO: n = 6, 3 litters; control LF: n = 5, 3 litters; control HFL: n = 6, 3litters; control HFO: n = 6, 6 litters.

Since dietary fat composition clearly alters diabetes incidence in Swiss Webster mice, we took a targeted plasma metabolomics approach to further explore effects of diet and early overnutrition on lipid regulation. Analysis of metabolomics data ranking plasma metabolites by Variable Importance in Projection (VIP) score, found that 9 of the top 15 scores were in phosphatidylcholines (Suppl. Fig. S2a). A heat map of phosphatidylcholines compared by diet and condition (Suppl. Fig. S2b) showed that much of the variation was a factor of diet. However, a number of phosphatidylcholines differed between control and early overnutrition mice within the HFO diet. Since we were most struck by the observation that HFO diet increased diabetes incidence in early overnutrition mice but decreased it in control mice, we were particularly interested in any metabolite differences that could contribute to this difference. Further analysis of phosphatidylcholines found that 6 had significant group by diet interaction effects (Fig. 3, ANOVA details in Supplementary Table S1). Tukey post hoc analysis showed reduced phosphatidylcholines in early overnutrition HFO mice relative to HFL counterparts or control HFO mice in 5 of 6 of the metabolites (PC ae C32:1, PC aa C36:1, PC ae C38:1, PC ae C38:2, PC ae C38:5). Post hoc analysis did not reach statistical significance for PC ae C30:1. Notably, observed differences were in phosphatidylcholine metabolites containing ultra long chain fatty acids (≥ C26:0).

### Plasma phosphatidylcholines are altered by diabetes

**Figure 4:**
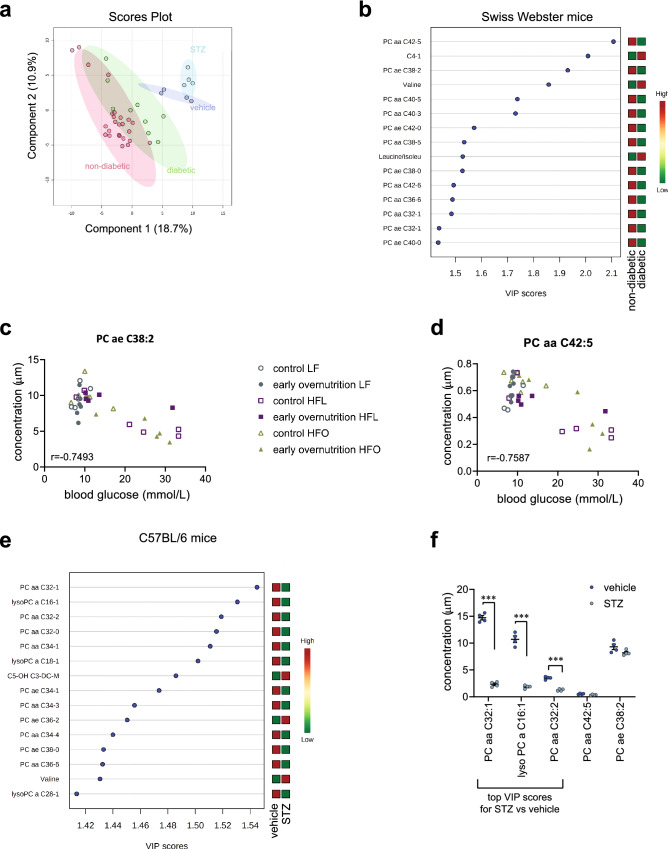
**(a)** Scores plot for principal component analysis comparing metabolites among diabetic streptozotocin (STZ)- or non-diabetic vehicle-treated C57BL/6 mice and diabetic and non-diabetic Swiss Webster mice. **(b)** Top 15 VIP scores that distinguish diabetic vs non-diabetic Swiss Webster mice. Heat map to right represents relative concentration of each metabolite. Two phosphatidylcholines, **(c)** PC ae C38:2 and **(d)** PC aa C42:5, showed the highest correlation to blood glucose in Swiss Webster mice tested by Pearson correlation, with p < 0.0001 for both. **(e)** Top 15 VIP scores that distinguish STZ- and vehicle-treated C57BL/6 mice with the concentrations of the top 3 scores plotted in **(f)**. ***Significantly reduced in STZ vs vehicle, p < 0.0001. *LF* low-fat diet; *HFL* high-fat lard-based diet; *HFO* high-fat olive oil/fish oil-based diet. Early overnutrition LF: n = 6, 3 litters; early overnutrition HFL: n = 5, 3 litters; early overnutrition HFO: n = 6, 3 litters; control LF: n = 5, 3 litters; control HFL: n = 6, 3litters; control HFO: n = 6, 6 litters. STZ and vehicle, n = 4.

As some mice were diabetic at the time of metabolomics analysis (at P70), including control and early overnutrition mice in both HFL and HFO diet groups, we next explored the effect of diabetes in Swiss Webster mice on plasma metabolites. We also explored another model of diabetes, the streptozotocin (STZ) -treated C57BL/6 mouse, to determine whether any alterations could be generalized to hyperglycemia or whether profiles were distinct between the two models. Samples were taken at 99 days post-STZ treatment, when fasting glucose values were 25.8 (21.8–27.2) mmol/L (median, interquartile range) and vehicle-treated glucose levels were 7.8 (7.0–8.3) mmol/L. A scores plot (Fig. 4a) of all metabolites showed no overlap between the STZ- and vehicle-treated C57BL/6 mice and Swiss Webster diabetic and non-diabetic mice. In Swiss Webster mice, 12 of the top 15 VIP scores that distinguish non-diabetic and diabetic Swiss Webster mice were phosphatidylcholine metabolites (Fig. 4b), and in all cases the phosphatidylcholines were reduced by diabetes. PC ae C38:2 and PC aa C42:5 showed the strongest negative correlation with blood glucose levels (Fig. 4c,d). However, these metabolites did not differ significantly between STZ- and vehicle-treated mice and there was little overlap in top 15 VIP scores with Swiss Webster mice, with phosphatidylcholines representing 13 of 15 top metabolites and all but PC ae C36:2 were decreased by diabetes (Fig. 4e,f).

### Acylcarnitines, sphingomyelins, and amino acids

Acylcarnitines and sphingomyelins varied mainly by diet (Suppl Fig. S3) among Swiss Webster mice. While STZ-treatment significantly lowered glutamine levels and increased the branched chain amino acids (BCAA’s) valine and leucine/isoleucine, these and other amino acids did not vary with diet or early overnutrition in Swiss Webster mice. BCAA’s were also not affected by diabetes status in Swiss Webster mice, with valine levels (median, interquartile range) of 252.8 (225.1–291.7) µM in non-diabetic and 275.3 (228.6–322.7) µM in diabetic mice, and leucine/isoleucine levels of 294.4 (248.0–350.2) µM in non-diabetic and 312.7 (256.1–393.0) µM in diabetic mice.

## Discussion

We previously showed that a combination of early overnutrition and a high-fat, lard-based diet increased diabetes incidence in Swiss Webster mice, even when the diet exposure was limited to the preweaning period^6^. Here we explored whether a high fat diet with a presumably healthier fatty acid profile of olive oil and fish oil could reduce diabetes incidence in early overnutrition mice. However, we surprisingly found that diabetes incidence and metabolic outcome were worse with the olive and fish oil-based diet compared to the lard-based diet on a background of early overnutrition. In addition, early overnutrition led to persistent alterations in plasma metabolomics, particularly in phosphatidylcholine levels with HFO diet. In contrast, control mice that were not exposed to early overnutrition had lower diabetes incidence with HFO diet. Thus, adverse effects of early overnutrition offset any potential metabolic benefit of the HFO diet.

Early overnutrition pups exhibit increased milk intake during the first two weeks of life compared to control pups^6^. Total fat content of maternal milk is typically maintained at a high level (~ 55 kcal% fat in mice) and milk fatty acid composition is not a strict reflection of dietary fat intake. However, increased intake of some dietary fats does translate to altered milk composition^20–22^. For instance, maternal olive oil consumption has been shown to increase milk content of oleic acid, the main component of olive oil, resulting in reduced body weight gain in offspring^23^. Maternal fish oil consumption increases milk content of omega-3 polyunsaturated fatty acids, particularly DHA, which is beneficial for development and reduces adiposity in offspring^21^. Thus, although we did not analyze milk fat composition in this study, we expect that the HFO diet would increase pup intake of oleic acid and omega-3 fats and reduce weight gain and adiposity in pups compared to HFL pups, particularly during the first two weeks of life when pup nutrient intake is exclusively from maternal milk. Unexpectedly, early overnutrition pups of HFO-consuming dams gained even more weight and had even higher adiposity at weaning compared to pups of HFL-consuming dams. Since milk intake in young pups is largely regulated by availability, we do not believe that the higher weight gain with early overnutrition HFO pups compared to HFL is due to higher milk intake but rather a specific effect of the different dietary fat profile on growth and adipogenesis. This adverse early development translated to long-term metabolic dysregulation. On the other hand, control pups showed the expected attenuation in high-fat diet-induced body weight gain and adiposity in early life suggesting that the dietary composition has the potential to improve metabolic health, but this was negated by early overnutrition.

In adulthood, early overnutrition HFO offspring no longer had higher body weight or adiposity relative to HFL counterparts, although early overnutrition males remained moderately heavier with higher adiposity and higher liver weight relative to control males. Although we did not measure food intake in adults, we previously showed similar chow intake in early overnutrition and control mice postweaning^6^. Surprisingly, HFO diet did not reduce diabetes incidence in early overnutrition mice compared to HFL diet, but actually increased incidence, with all early overnutrition HFO mice developing diabetes by 6 months of age. The accelerated body weight gain and adiposity that were already observed in early overnutrition HFO pups by weaning suggest that the adverse effects of this diet may have occurred early in life. In contrast, in control mice diabetes incidence was lower with HFO compared to HFL diet, although this did not reach statistical significance. As we have previously seen^6^, early overnutrition LF fed mice had the lowest diabetes incidence indicating that early overnutrition alone is only detrimental with higher dietary fat content. Although control HFO mice had a lower diabetes incidence, they displayed glucose intolerance at P59, as did early overnutrition mice suggesting that while the diet may protect against beta-cell failure, glucose dysregulation may still occur. However, at P70 we observed high insulin levels in control HFO mice suggesting that their beta-cells were able to mount a compensatory response which may have prevented them from progressing to beta-cell failure and diabetes. We previously showed that diabetes in Swiss Webster mice is not secondary to insulin resistance but is due to beta-cell dysfunction and death promoted by early overnutrition on moderate and high fat lard-based diet. Similarly, in the present study diabetes did not appear to be secondary to insulin resistance since insulin sensitivity was similar in all groups. However, a higher insulin dose or euglycemic-hyperinsulinemic clamp would assess this more definitively. Moreover, insulin levels were elevated in early overnutrition pups relative to control but were no longer elevated by adulthood, even in the face of hyperglycemia, suggesting a failure of beta-cells to properly respond to increased metabolic demand. Similarly, although we did not observe a significant difference in beta-cell mass at P70 in early overnutrition HFO and HFL mice relative to LF mice, the failure to increase beta-cell mass in the face of the higher metabolic demand of a high fat diet may have contributed to their increased diabetes susceptibility.

Although numerous studies suggest overall beneficial metabolic effects of olive oil and fish oil^24–27^, some adverse effects have been shown with exposure to high doses of fish oil or omega-3 fats during prenatal and postnatal development, particularly low body weight and developmental delay^28,29^. However, we did not observe such effects with our diet and instead saw higher body weight and adiposity with early overnutrition on the HFO diet relative to HFL diet. Detrimental effects of high dose omega-3 fats seen in other studies may be partly due to low relative omega-6 levels since the ratio of omega-6 to omega-3 is critical for maintaining optimal health^30^. The ratio in our HFO diet was 3:1, which is in the range of a Mediterranean-style diet and close to the optimal ratio of 1:1 to 2:1^30^, compared to 8.5:1 in the HFL diet. Therefore, we do not believe that excess developmental omega-3 exposure contributed to the higher diabetes incidence in our early overnutrition mice. Fish oil also made up only a small portion (3%) of the HFO diet, with olive oil as the main source of fat (15%). A dietary shift favouring oleic acid over saturated fats has been shown to improve insulin sensitivity in humans^31^. However, since diabetes in Swiss Webster mice appears to be due to beta-cell dysfunction that is not secondary to insulin resistance, excess HFO intake in early life may have adversely affected long-term beta-cell function and survival.

Numerous studies have demonstrated that chronic elevations of the saturated fat palmitic acid can have detrimental effects on beta-cells, including promotion of endoplasmic reticulum stress, oxidative stress, and apoptosis, as well as reduced proliferation^12^. Oleic acid has been shown to attenuate some of these detrimental effects and is more effective in stimulating insulin secretion and proliferation^12^. Thus, we expected the HFL diet to improve beta-cell function and proliferation in response to the metabolic demands of a high-fat diet. Although this may have been the case in control mice, early overnutrition mice did not show this response. However, there is some evidence that oleic acid may have similar lipotoxic effects as palmitic acid in beta-cells^13^. Since beta-cells of Swiss Webster mice are highly susceptible to high fat diet-mediated dysfunction^6^, excess intake of oleic acid in early life may have impaired normal beta-cell development rendering the mice more susceptible to the metabolic stress of continued high fat diet exposure. In addition, since early overnutrition mice had higher adiposity on the HFO diet at a young age, a higher degree of dyslipidemia may have contributed to their higher diabetes incidence relative to HFL offspring. However, the combination of both olive oil and fish oil in our HFO diet limits our ability to address the effect of specific fatty acids on metabolic development.

Targeted plasma metabolomics in adulthood revealed several diet-induced lipid differences, as expected. In addition, early overnutrition induced some long-term alterations in metabolites, with particular divergence in HFO mice in phosphatidylcholines with ultra long chain fatty acids. Phospholipid composition of membranes is important for cell function, with fatty acid chain length and saturation affecting cell signaling^32^. Whether early overnutrition mediated alterations in phosphatidylcholines contributed to glucose dysregulation in HFO mice is unclear. However, several previous studies have found reductions in plasma phosphatidylcholines associated with impaired glucose tolerance and diabetes in rodents^33^ and humans^33–36^, although phosphatidylcholine species examined and altered by diabetes are heterogeneous among studies. Reduced plasma phosphatidylcholine levels in our study were also seen in normoglycemic early overnutrition HFO mice, and all mice exhibited similar insulin sensitivity, suggesting that alterations were not a secondary response to hyperglycemia or insulin resistance. Although we did not measure phosphatidylethanolamine levels in this study, an elevated phosphatidylcholine to phosphatidylethanolamine ratio has previously been observed in hepatic ER of obese mice, with improved glucose homeostasis upon normalization of the ratio^37^. Thus, the relative composition of different phospholipid species can have an important impact on metabolism. The metabolomic profile of diabetic Swiss Webster mice also differed from that of STZ-induced diabetes in C57BL/6 mice, reflecting the different etiology of diabetes development between these two models, with the former being a high fat diet-induced beta-cell death whereas the latter is a toxin-induced beta-cell death. However, the longer duration of diabetes in STZ mice relative to the Swiss Webster mice may have also contributed to differences since long-term lipolysis would result in altered lipid profiles and hepatic lipid handling. Furthermore, diabetic Swiss Webster mice included mice from both control and early overnutrition groups that developed diabetes either on the HFL or HFO diets; thus, the differing backgrounds may have affected the ultimate diabetic phenotype. Reductions in phosphatidylcholines were also predominant in STZ-induced diabetes, although the species affected differed from diabetic Swiss Webster mice. In addition, STZ increased BCAA levels whereas levels did not differ in Swiss Webster mice in response to diet, early overnutrition, or by diabetes.

The present study has several limitations. Since the metabolomics we performed targeted only select phosphatidylcholines and we examined plasma and not tissues, we do not have a complete picture of phospholipid composition to assess how and where signalling may be altered. However, given the primary beta-cell dysfunction in Swiss Webster mice^6^ as well as the unexpected effects of the HFO diet on adiposity and leptinemia, tissue specific examination of phosphatidylcholines in beta-cells and adipocytes, and their role in glucose and lipid regulation, is warranted. To avoid possible programming effects due to excess lipid exposure during fetal development, pregnant dams were maintained on the LF diet during pregnancy. However, it is possible that low dietary fat content during development may have had a detrimental effect on fetal development. Thus, further exploration of the impact of low and high dietary fat intake during pregnancy on fetal development in Swiss Webster mice is merited. Since detrimental effects of the diets likely began during the suckling period, analysis of milk composition is important to fully understand the dietary fat profile that pups were exposed to. Finally, the combination of both olive oil and fish oil in the HFO diet limits our ability to assess the lipotoxic versus protective effects of the individual dietary fat sources.

The present study suggests that beneficial effects of a Mediterranean style dietary fat composition, favouring monounsaturated over saturated fats and a low omega-6:omega-3 ratio, may be diminished by overconsumption in early life and there may be detrimental effects on beta-cell function and survival. Thus, consideration should be taken in not only the fatty acid composition of a diet but also the overall level of intake and the timing of diet with adverse effects of diet more likely during early life when metabolic tissues are undergoing significant development. In addition, a relationship between reduced phosphatidylcholine species and impaired glucose regulation was observed. Whether these alterations can serve as biomarkers of diabetes progression and whether they may contribute to the development of diabetes requires further investigation.

## Methods

### Animals and diet

All mice were maintained on a 12 h/12 h light–dark cycle with constant temperature and humidity and ad libitum access to food and water. All animal procedures were approved by the University of British Columbia Animal Care Committee (protocol #A17-0026), carried out in accordance with the Canadian Council of Animal Care guidelines, and complied with ARRIVE guidelines. For breeding, Swiss Webster male and female mice were purchased from Taconic (Taconic, Germantown, NY) and placed on LF diet (#D12450B, Research Diets, Inc., New Brunswick, NJ) upon arrival at 6 weeks of age. Mice were pair-mated at 7 weeks of age and females were maintained on LF diet until 2 days after delivery, designated as postnatal day (P) 2. At this time, dams either remained on LF diet, were switched to HFL diet (45 kcal% fat, #D12451, Research Diets) or HFO diet (45 kcal% fat; custom diet #D10072204, Research Diets). The HFO diet was formulated to provide a calorie matched high-fat diet with preference for monounsaturated over saturated fats and increased omega-3 levels compared to the HFL diet. Diet composition and fatty acid profiles of the three diets are in Tables 1 and 2.

For control offspring, litters were adjusted to 10 pups per litter at P2. For the early overnutrition group, litters were initially adjusted to 6 pups per litter and further reduced to 3 pups per litter at P5, since earlier reduction to 3 pups may lead to inadequate maternal milk production. Upon weaning at P21, offspring were maintained on the same diet as the dams during lactation. Since we previously found that only male Swiss Webster mice were prone to diabetes^6^, all experiments were performed in male offspring only. C57BL/6 mice were obtained from the University of British Columbia Centre for Disease Modeling. Mice were injected intraperitoneally with 180 mg/kg STZ to render them diabetic. Age-matched vehicle-treated control mice were injected with acetate buffer, pH 4.5. Blood samples were collected at 99 days post-injection at which time STZ mice remained diabetic.

### Glucose monitoring and tolerance tests

Beginning at P30, 4 h fasted blood glucose was measured weekly from the saphenous vein using a OneTouch Ultra 2 glucometer (LifeScan, Inc., Burnaby, Canada), with diabetes defined as a reading ≥ 20 mmol/L. For oral glucose tolerance tests, mice were fasted for 6 h beginning at lights on. A basal blood glucose measure was taken and ~ 20 μL of blood collected via the saphenous vein using a heparinized capillary tube. Mice were then gavaged with glucose at 2 g/kg body weight and additional blood glucose measures and blood samples were collected at 7, 15, and 60 min relative to glucose gavage. Plasma was stored at -30 °C prior to assay for insulin. For insulin tolerance tests, mice were fasted for 4 h beginning at 2 h after lights on. Mice were then injected intraperitoneally (ip) with 0.75 U/kg body weight insulin (Novolin ge Toronto, Novo Nordisk Canada, Mississauga, Canada). Blood glucose was measured just prior to insulin injection (0 min), and at 10, 20, 30, 60, 90, and 120 min post-injection.

### Blood collection and tissue harvest

For terminal measures, P70 mice were first fasted for 4 h while P21 mice were non-fasted. A blood glucose measure was taken from the saphenous vein. Mice were then sedated with isoflurane and a cardiac blood sample was collected for measurement of hormones by ELISA. Whole fat pads and liver were removed and weighed on an analytical balance, post-fixed in 4% paraformaldehyde for 24 h, and then transferred to 70% ethanol prior to paraffin-embedding and sectioning at 5 μm by Wax-it Histology Services Inc. (Vancouver, Canada). Plasma insulin was measured using the ALPCO Mouse Ultrasensitive Insulin ELISA (Salem, NH) and leptin was measured using the Crystal Chem Mouse Leptin ELISA (Elk Grove Village, IL).

### Immunohistochemistry

Immunohistochemistry on pancreas sections was performed as previously described^6^ using rabbit monoclonal insulin (#3014, RRID:AB_2126503, 1:200, Cell Signaling, Danvers, MA) and mouse monoclonal glucagon (#G2654, RRID:AB_259852, 1:1000, Millipore Sigma, Burlington, MA) primary antibodies, with donkey anti-rabbit 594 and donkey anti-mouse 488 secondary antibodies (Life Technologies, Carlsbad, CA). For quantification of beta-cell mass, slides were scanned on an ImageXpress Micro imaging system and the thresholded area of insulin-positive signal was quantified using MetaXpress software. Three sections per mouse, 200 μm apart, were analyzed for each sample. Beta-cell mass was calculated by multiplying the average ratio of insulin-positive area over total pancreas area by total pancreas weight. Islet number was calculated as mean number of islets per pancreas section averaged across the three sections per mouse.

### Metabolomics and statistical analyses

Targeted metabolites were analyzed using the AbsoluteIDQ p180 Kit from Biocrates Life Sciences AG (Innsbruck, Austria) in plasma collected after a 4 h fast from P70 Swiss Webster mice as well as C57BL/6 STZ- and vehicle-treated mice. Metabolites measured include 15 lysophosphatidylcholines, 77 phosphatidylcholines, 14 amino acids, 15 sphingomyelins, 41 acylcarnitines, and total hexoses. All metabolite concentrations were first analyzed using Metaboanalyst 4.0 Statistical Analysis^38^, with normalization by sum plus autoscaling. Partial least squares—discriminant analysis was used to determine the top 15 metabolites ranked by variable importance in projection (VIP) score. All phosphatidylcholines were further analyzed by Metaboanalyst two-factor independent sample analysis and a heatmap generated using Euclidian distance measure and the complete clustering algorithm. ANOVA Simultaneous Component Analysis (ASCA)^39^ was used to identify main and interaction effects among metabolites.

Statistical analyses for tissue weights, glucose, and insulin data were performed using GraphPad Prism (San Diego, CA) by two-way ANOVA or two-way repeated measures ANOVA as indicated, with Tukey post-hoc correction for multiple comparisons and significance defined as p < 0.05. F-test results for main and interaction effects are listed in Tables 3, 4, and Supplementary Table S1. Diabetes incidence was assessed by log-rank tests.

## Supplementary Information

Supplementary Information.

## Data Availability

Metabolomics data has been deposited on figshare: https://figshare.com/s/14aa1f9301865aead95b.
