# Supplementary material for: Early overnutrition in male mice negates metabolic benefits of a diet high in monounsaturated and omega-3 fats
Source: Sci Rep. 2021 Jul 7;11:14032. doi: 10.1038/s41598-021-93409-z (PMC8263808; doi:10.1038/s41598-021-93409-z)
Supplement: Supplementary file 1 — Supplementary Information. [file 41598_2021_93409_MOESM1_ESM.pdf]

SUPPLEMENTARY MATERIAL

Early overnutrition in male mice negates metabolic benefits of a diet high in monounsaturated and omega-3 fats

Maria M. Glavas[1], Queenie Hui[1], Ian Miao[1], Fan Yang[1], Suheda Erener[1], Kacey J. Prentice[2], Michael B. Wheeler[2,3], Timothy J. Kieffer[1,4,5*]

[1]Department of Cellular and Physiological Sciences, University of British Columbia, Vancouver, BC, Canada; [2]Department of Physiology, University of Toronto, Toronto, Canada; [3]Department of Advanced Diagnostics, Toronto General Hospital Research Institute, University Health Network, Toronto, Canada; [4]Department of Surgery, University of British Columbia, Vancouver, BC, Canada

[5]School of Biomedical Engineering, University of British Columbia, Vancouver, BC, Canada

***Corresponding Author:**

Timothy J. Kieffer

Department of Cellular and Physiological Sciences, 2350 Health Sciences Mall Rm 5320, University of British Columbia, Vancouver, BC, Canada, V6T 1Z3

Tel +1 604 822 2156

Email: tim.kieffer@ubc.ca

Supplementary Table 1. Two-way ANOVAs corresponding to figure panels.

Figure Panel	Measure	Source of Variation	MS	F (DFn, DFd)	p-value
Fig. 1b	P2-15 weight gain	Group	496.9	581.7 (1,160)	<0.0001*
		Diet	29.86	34.95 (2,160)	<0.0001*
		Group x Diet	17.49	20.48 (2,160)	<0.0001*
Fig. 1c	EWAT weight	Group	0.235	85.53 (1,52)	<0.0001*
		Diet	0.045	16.45 (2,52)	<0.0001*
		Group x Diet	0.013	4.57 (2.52)	0.015*
Fig. 1d	mWAT weight	Group	0.052	45.91 (1,52)	<0.0001*
		Diet	0.020	17.40 (2,52)	<0.0001*
		Group x Diet	0.006	5.59 (2,52)	0.006*
Fig. 1e	P21-47 weight gain	Group	8.54	1.40 (1,90)	0.240
		Diet	45.65	7.48 (2,90)	0.001*
		Group x Diet	2.72	0.44 (2,90)	0.643
Fig. 1g	Glucose response	Condition	382	2.89 (5,67)	0.0202*
		Time	1722	143.9 (2.3, 150.8)	<0.0001*
		Condition x Time	20.56	1.72 (15, 201)	0.0496*
Fig. 1h	Insulin response	Condition	29.58	2.16 (5, 67)	0.0691
		Time	7.05	5.98 (2.59, 173.8)	0.0012*
		Condition x Time	1.70	1.44 (15, 201)	0.131
Fig. 1j	Glucose during ITT	Condition	686.2	0.65 (5, 53)	0.6627
		Time	11764	92.67 (3.83, 203)	<0.0001*
		Condition x Time	208.5	1.64 (30, 318)	0.0208*
Fig. 1k	ITT area under curve	Group	3917730	1.31 (1, 53)	0.2580
		Diet	1555776	0.52 (2, 53)	0.5979
		Group x Diet	516006	0.17 (2, 53)	0.8422
Fig. 2b	Beta-cell mass	Group	1.57	0.57 (1, 18)	0.4577
		Diet	0.89	0.33 (2, 18)	0.7266
		Group x Diet	7.53	2.76 (2, 18)	0.0902
Fig. 2c	Islet number	Group	98.69	0.27 (1, 18)	0.6093
		Diet	256.6	0.70 (2, 18)	0.5081
		Group x Diet	582.4	1.60 (2, 18)	0.2300
Fig. 3a	PC ae C30:1	Group	0.00126	0.32 (1, 28)	0.5747
		Diet	0.00783	2.01 (2, 28)	0.1526
		Group x Diet	0.0243	6.23 (2, 28)	0.0058*
Fig. 3b	PC ae C32:1	Group	0.00179	0.044 (1, 28)	0.8351
		Diet	0.522	12.84 (2, 28)	0.0001*
		Group x Diet	0.352	8.66 (2, 28)	0.0012*
Fig. 3c	PC aa C36:1	Group	444	1.26 (1, 28)	0.2706
		Diet	334.9	0.95 (2, 28)	0.3978
		Group x Diet	2075	5.90 (2, 28)	0.0073*
Fig. 3d	PC ae C38:1	Group	0.0553	0.140 (1, 28)	0.7108
		Diet	0.597	1.516 (2, 28)	0.2371
		Group x Diet	2.673	6.788 (2, 28)	0.0039*
Fig. 3e	PC ae C38:2	Group	5.38	1.34 (1, 28)	0.2577
		Diet	4.79	1.19 (2, 28)	0.3197
		Group x Diet	31.27	7.75 (2, 28)	0.0021*
Fig. 3f	PC ae C38:5	Group	4.03	2.44 (1, 28)	0.1293
		Diet	15.41	9.34 (2, 28)	0.0008*
		Group x Diet	12.16	7.37 (2, 28)	0.0027*
Fig. 4 f	STZ top VIP scores	Treatment	246.1	641.6 (1, 30)	<0.0001*
		Metabolite	110.9	289.0 (4, 30)	<0.0001*
		Treatment x Metabolite	57.96	151.1 (4, 30)	<0.0001*

Group: early overnutrition vs control; Diet: LF vs HFL vs HFO. Condition: early overnutrition LF, HFL, HFO, control LF, HFL, HFO. Treatment: streptozotocin (STZ) vs vehicle. P=postnatal; EWAT = epididymal white adipose tissue; mWAT: mesenteric white adipose tissue; ITT = insulin tolerance test; DFn, DFd = degrees of freedom of numerator and denominator of the F ratio. *Significant main or interaction effect by two-way ANOVA (two-way repeated measures ANOVA for Fig. 1g, h, j).

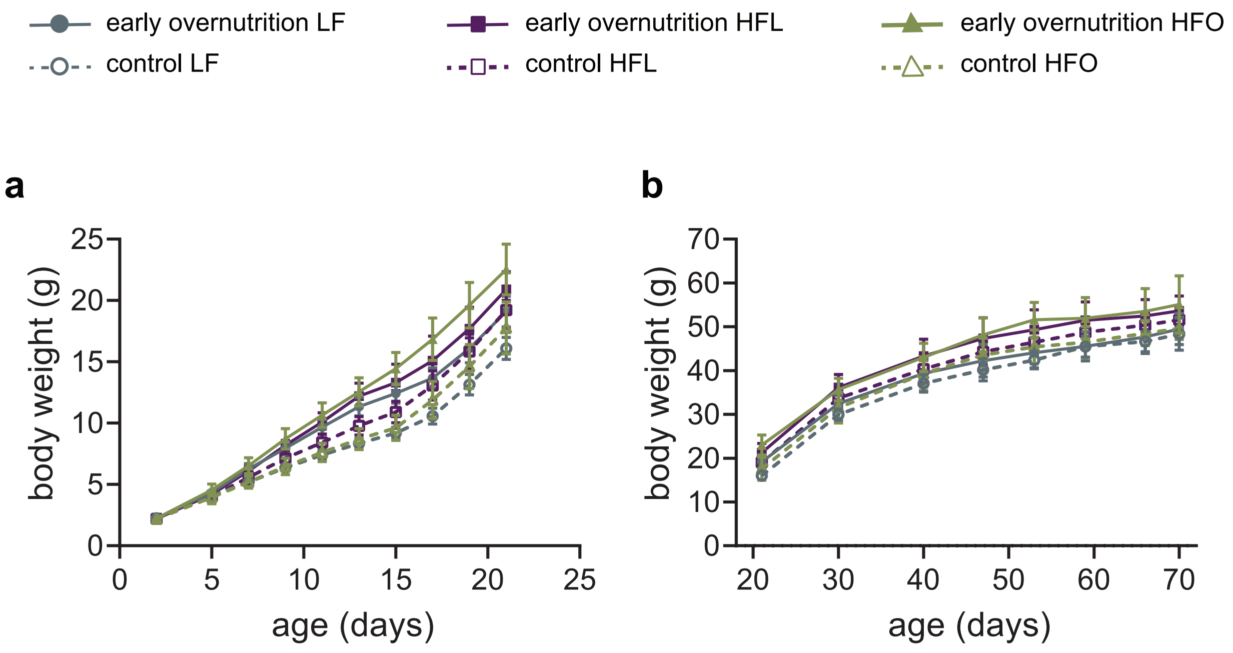

Supplemental Figure 1. Body weight gain from **(a)** postnatal day (P) 2 to 21, and **(b)** P21 to 70. Data represent mean ± SEM with n = control LF: 26; early overnutrition LF: 21; control HFL: 29; early overnutrition HFL: 20; control HFO: 38; early overnutrition HFO: 27 in panel **(a)** and n= control LF: 9; early overnutrition LF: 9; control HFL: 10; early overnutrition HF: 10; control HFO: 9; early overnutrition HFO: 8 in panel **(b)**. LF: low-fat diet (10 kcal% fat); HFL: high-fat lard-based diet (45 kcal% fat); HFO: high-fat olive oil/fish oil-based diet (45 kcal% fat).

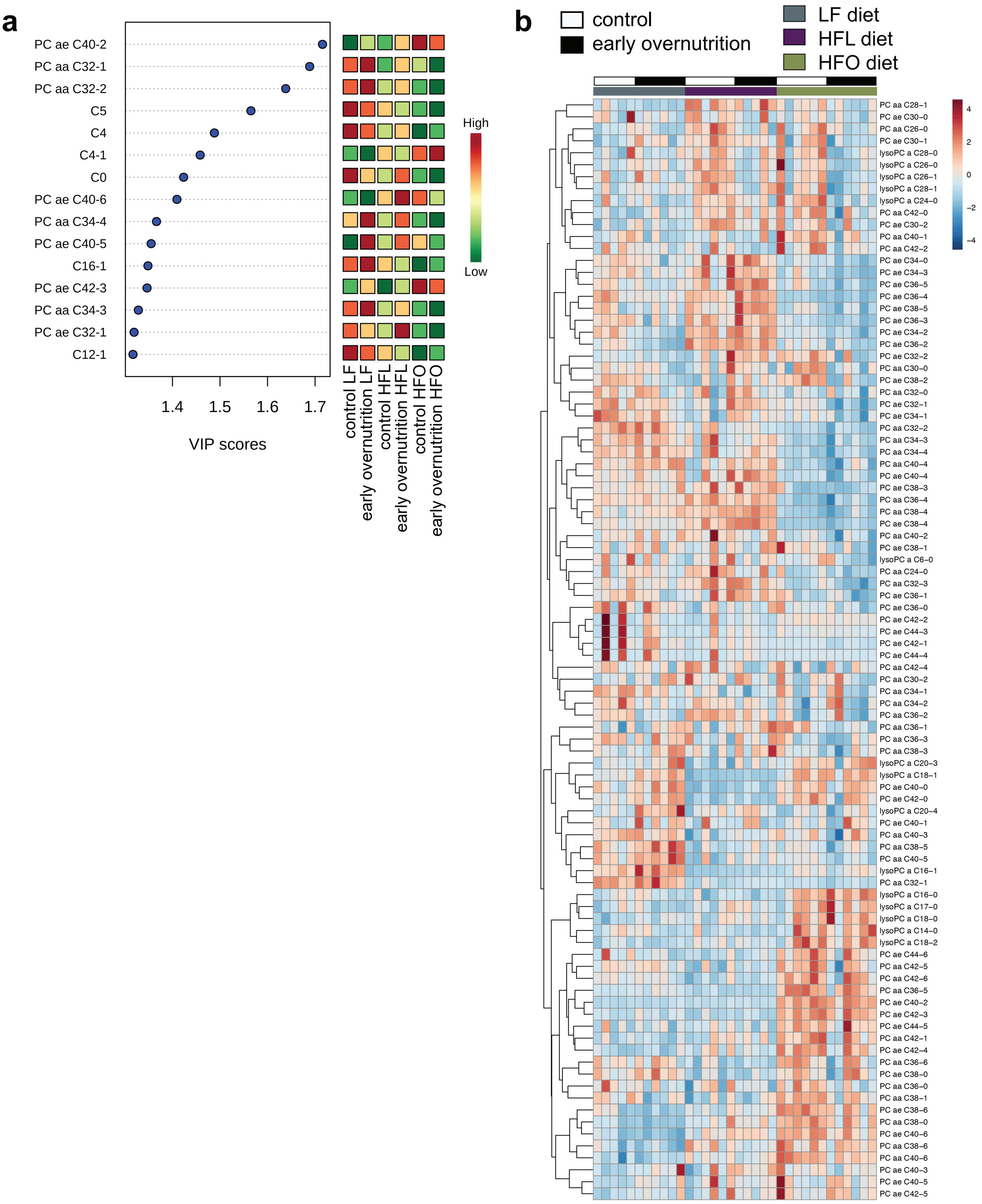

Supplementary Figure 2. (a) Top 15 metabolites ranked by Variable Importance in Projection (VIP) . Red-green heat map on the right compares relative concentrations for each metabolite. **(b)** Heat map of phospholipids among treatment and diet conditions. Images generated with Metaboanalyst 4.0.

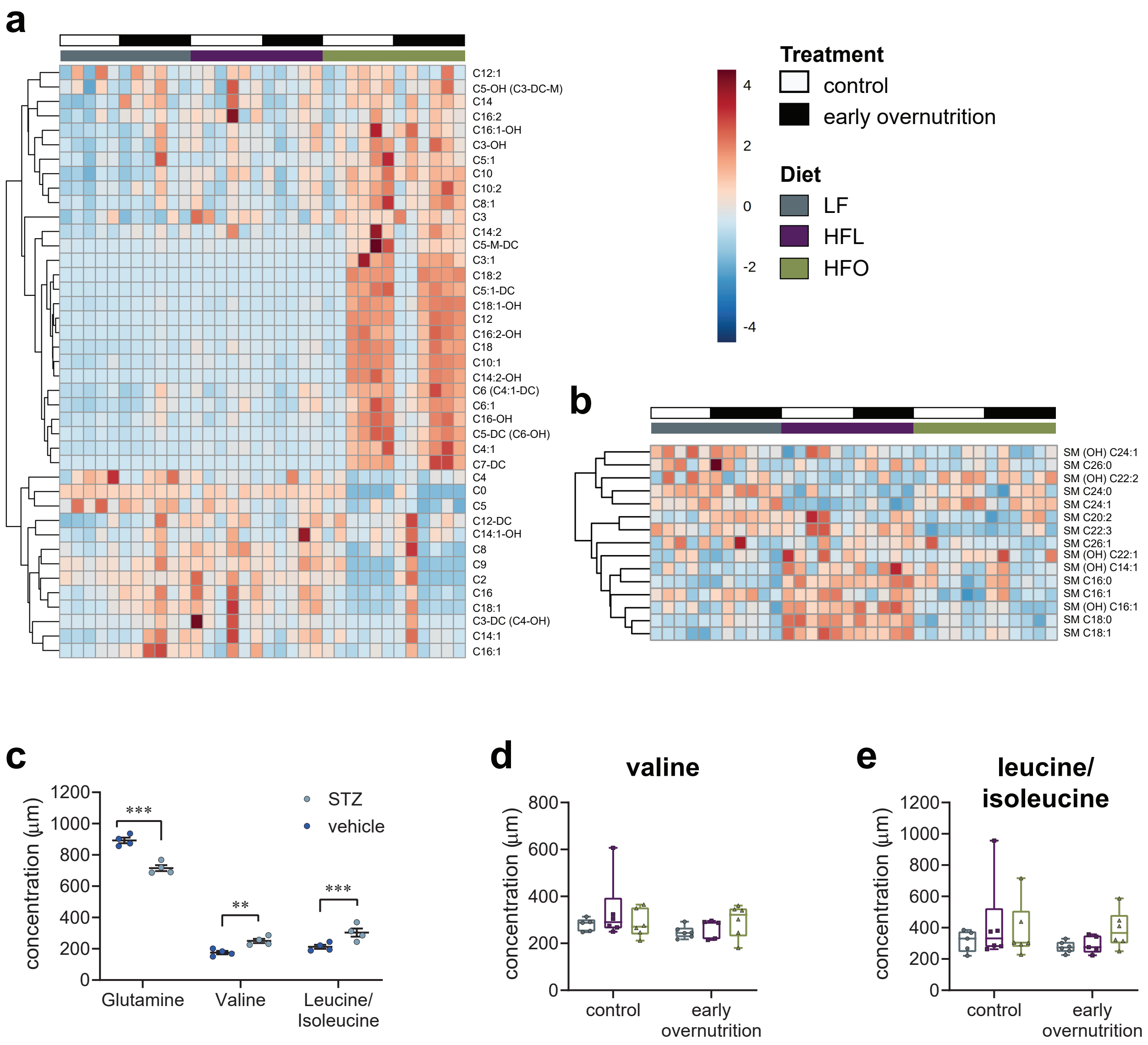

Supplementary Figure 3. Heat maps of **(a)** acylcarnitines and **(b)** sphingomyelins in control and early overnutrition male mice at postnatal day (P) 70 maintained on low-fat diet (LF), lard-based high-fat diet (HFL) or olive oil/fish oil-based high-fat diet (HFO), generated with Metaboanalyst 4.0. Observed differences are mainly related to diet. **(c)** STZ-treated mice exhibited reduced glutamine and increased branched-chain amino acids (BCAA's, valine and leucine/isoleucine) relative to vehicle-treated mice (**$p<0.01$; ***$p<0.0001$). In Swiss Webster mice the BCAA's **(d)** valine and **(e)** leucine/isoleucine did not differ by postnatal treatment or diet and no differences were observed in any other amino acids. LF: 10 kcal% fat diet; HFL: 45 kcal% fat lard-based diet; HFO: 45 kcal% fat olive oil/fish oil-based diet; STZ: streptozotocin.
